# Impact of weight loss on cancer-related proteins in serum: results from a cluster randomised controlled trial of individuals with type 2 diabetes

**DOI:** 10.1016/j.ebiom.2024.104977

**Published:** 2024-01-29

**Authors:** Caroline J. Bull, Emma Hazelwood, Danny N. Legge, Laura J. Corbin, Tom G. Richardson, Matthew Lee, James Yarmolinsky, Karl Smith-Byrne, David A. Hughes, Mattias Johansson, Ulrike Peters, Sonja I. Berndt, Hermann Brenner, Andrea Burnett-Hartman, Iona Cheng, Sun-Seog Kweon, Loic Le Marchand, Li Li, Polly A. Newcomb, Rachel Pearlman, Alex McConnachie, Paul Welsh, Roy Taylor, Mike E.J. Lean, Naveed Sattar, Neil Murphy, Marc J. Gunter, Nicholas J. Timpson, Emma E. Vincent

**Affiliations:** a MRC Integrative Epidemiology Unit at the University of Bristol, Bristol, UK; b Population Health Sciences, Bristol Medical School, University of Bristol, Bristol, UK; c School of Translational Health Sciences, Dorothy Hodgkin Building, University of Bristol, Bristol, UK; d Section of Nutrition and Metabolism, International Agency for Research on Cancer, WHO, Lyon, France; e Cancer Epidemiology Unit, Oxford Population Health, University of Oxford, UK; f Public Health Sciences Division, Fred Hutchinson Cancer Research Center, Seattle, WA, USA; g Division of Cancer Epidemiology and Genetics, National Cancer Institute, National Institutes of Health, Bethesda, MD, USA; h Division of Clinical Epidemiology and Aging Research, German Cancer Research Center (DKFZ), Heidelberg, Germany; i Division of Preventive Oncology, German Cancer Research Center (DKFZ) and National Center for Tumor Diseases (NCT), Heidelberg, Germany; j German Cancer Consortium (DKTK), German Cancer Research Center (DKFZ), Heidelberg, Germany; k Institute for Health Research, Kaiser Permanente Colorado, Denver, CO, USA; l Department of Epidemiology and Biostatistics, University of California, San Francisco, San Francisco, CA, USA; m Department of Preventive Medicine, Chonnam National University Medical School, Gwangju, Korea; n Jeonnam Regional Cancer Center, Chonnam National University Hwasun Hospital, Hwasun, Korea; o University of Hawaii Cancer Center, Honolulu, HI, USA; p Department of Family Medicine, University of Virginia, Charlottesville, VA, USA; q School of Public Health, University of Washington, Seattle, WA, USA; r Division of Human Genetics, Department of Internal Medicine, The Ohio State University Comprehensive Cancer Center, Columbus, OH, USA; s Robertson Centre for Biostatistics, Institute of Health and Wellbeing, University of Glasgow, Glasgow, G12 8QQ, UK; t School of Cardiovascular and Metabolic Health, University of Glasgow, Glasgow, UK; u Translational and Clinical Research Institute, Newcastle University, Newcastle upon Tyne, UK; v Human Nutrition, School of Medicine, Dentistry and Nursing, College of Medical, Veterinary & Life Sciences, University of Glasgow, Glasgow, UK; w Department of Epidemiology and Biostatistics, School of Public Health, Imperial College London, UK

**Keywords:** Weight loss, DiRECT, Diabetes, Obesity, Cancer, Mendelian randomization

## Abstract

**Background:**

Type 2 diabetes is associated with higher risk of several cancer types. However, the biological intermediates driving this relationship are not fully understood. As novel interventions for treating and managing type 2 diabetes become increasingly available, whether they also disrupt the pathways leading to increased cancer risk is currently unknown. We investigated the effect of a type 2 diabetes intervention, in the form of intentional weight loss, on circulating proteins associated with cancer risk to gain insight into potential mechanisms linking type 2 diabetes and adiposity with cancer development.

**Methods:**

Fasting serum samples from participants with diabetes enrolled in the Diabetes Remission Clinical Trial (DiRECT) receiving the Counterweight-Plus weight-loss programme (intervention, N = 117, mean weight-loss 10 kg, 46% diabetes remission) or best-practice care by guidelines (control, N = 143, mean weight-loss 1 kg, 4% diabetes remission) were subject to proteomic analysis using the Olink Oncology-II platform (48% of participants were female; 52% male). To identify proteins which may be altered by the weight-loss intervention, the difference in protein levels between groups at baseline and 1 year was examined using linear regression. Mendelian randomization (MR) was performed to extend these results to evaluate cancer risk and elucidate possible biological mechanisms linking type 2 diabetes and cancer development. MR analyses were conducted using independent datasets, including large cancer meta-analyses, UK Biobank, and FinnGen, to estimate potential causal relationships between proteins modified during intentional weight loss and the risk of colorectal, breast, endometrial, gallbladder, liver, and pancreatic cancers.

**Findings:**

Nine proteins were modified by the intervention: glycoprotein Nmb; furin; Wnt inhibitory factor 1; toll-like receptor 3; pancreatic prohormone; erb-b2 receptor tyrosine kinase 2; hepatocyte growth factor; endothelial cell specific molecule 1 and Ret proto-oncogene (Holm corrected *P*-value <0.05). Mendelian randomization analyses indicated a causal relationship between predicted circulating furin and glycoprotein Nmb on breast cancer risk (odds ratio (OR) = 0.81, 95% confidence interval (CI) = 0.67–0.99, *P*-value = 0.03; and OR = 0.88, 95% CI = 0.78–0.99, *P*-value = 0.04 respectively), though these results were not supported in sensitivity analyses examining violations of MR assumptions.

**Interpretation:**

Intentional weight loss among individuals with recently diagnosed diabetes may modify levels of cancer-related proteins in serum. Further evaluation of the proteins identified in this analysis could reveal molecular pathways that mediate the effect of adiposity and type 2 diabetes on cancer risk.

**Funding:**

The main sources of funding for this work were 10.13039/501100000361Diabetes UK, 10.13039/501100000289Cancer Research UK, 10.13039/501100000321World Cancer Research Fund, and 10.13039/100004440Wellcome.

**Research in context**

**Evidence before this study**

It is known that individuals with type 2 diabetes are at an increased risk of developing cancer. It is thought that increased adiposity may explain at least some of this risk, and previous studies have found that having an increased body weight alters the levels of certain proteins measured in blood (known as circulating proteins). Previous studies have also explored whether the altered levels of circulating proteins explain the increased cancer risk. However, few studies have evaluated the effect of weight-loss by individuals with type 2 diabetes on circulating proteins, or how the circulating proteins altered by weight loss relate to cancer risk.

**Added value of this study**

In this study, we investigated the impact of a weight-loss intervention on circulating proteins with a known link to cancer. We identified nine proteins which were altered following the intervention. We then evaluated these proteins for evidence of a causal role in increasing the risk of developing six obesity-driven cancers, using a genetic epidemiological method called Mendelian randomization.

**Implications of all the available evidence**

Based on all available evidence, it seems that weight management in individuals recently diagnosed with type 2 diabetes delivered in a primary care setting may influence biological pathways relevant to cancer, which may have implications for both policy implementation and future type 2 diabetes management research.

## Introduction

Type 2 diabetes is a chronic disease characterised by hyperinsulinemia and insulin resistance.^1^ Diabetes is on the rise globally; with prevalence having quadrupled between 1980 and 2017, it is estimated that over 600 million people could be afflicted worldwide by 2045.^2^ This mirrors the increase in obesity, which is the main modifiable risk factor for type 2 diabetes.^3^ First line treatment for type 2 diabetes usually comprises of oral hypoglycaemic agents, including metformin.^4^ However, recent research suggests that intentional weight-loss, even alongside withdrawal of antidiabetic and antihypertensive medication, may be at least as effective at achieving diabetes remission and restoration of normal insulin metabolism.^5^, ^6^, ^7^, ^8^, ^9^, ^10^

Type 2 diabetes is known to increase the risks of several diseases including certain cancers, in particular colorectal, breast, endometrial, gallbladder, liver and pancreatic cancer.^11^ However, the biological mechanisms thought to mediate the relationship between type 2 diabetes and cancer have not been fully elucidated. Additionally, the efficacy of weight-loss in a cohort of people with type 2 diabetes in reducing risk of cancer is unknown. We and others have shown that increased body mass index (BMI), which is closely linked to type 2 diabetes incidence, is associated with widespread metabolomic and proteomic alterations.^12^, ^13^, ^14^, ^15^, ^16^, ^17^, ^18^, ^19^ Perturbations in circulating biomarkers induced by obesity and diabetes may result in long-term systemic exposure of cells and tissues to an abnormal and dysregulated metabolic environment to which tumours must adapt to satisfy the bioenergetic and biosynthetic demands of chronic cell proliferation.^20^^,^^21^ Circulating biomarkers are therefore plausibly linked to the likelihood of cancer developing and to the characteristics of the resulting tumour.

A systematic review of weight-loss trials reported a significant reduction in the risk of cancer mortality, highlighting the success of these interventions for cancer prevention.^22^ However, whether weight-loss can mitigate the increased cancer risk seen in individuals with type 2 diabetes has not been extensively studied. The collection of blood samples in diabetes intervention trials permits the assessment of intermediate endpoints (e.g., molecular traits such as proteins) which may be associated with disease status or progression toward a secondary endpoint (e.g., cancer). Further, using Mendelian randomization (an epidemiological technique whereby genetic variants are used to proxy exposures of interest) it is possible (given specific assumptions) to estimate a potential causal relationship between circulating proteins, identified in the examination of trial data, and disease using *cis* protein quantitative trait loci (pQTLs).^23^^,^^24^ In this study, we set out to leverage the methodological properties of a weight-loss trial and to focus on circulating proteins, which can inform tissue crosstalk and physiological status, and which have the potential to increase understanding as to possible pathways connecting variation in adiposity, type 2 diabetes, and disease. Proteins may be particularly interesting factors to consider in this regard as they represent over 90% of drug targets.^25^ Proteins for which a causal role in cancer aetiology can be established may represent intervention targets to reduce disease risk.

The primary aim of this study was to identify proteins that were altered following intentional weight loss in a primary care randomised controlled trial framework (for trial protocol see).^26^ The causal relationship between intervention-associated proteins and six obesity-driven cancers was subsequently evaluated using genetic epidemiological methods.^27^

## Methods

### Study design and participants

Samples and clinical data were obtained from 313 participants of the Diabetes Remission Clinical Trial (DiRECT, ISRCTN registry number 03267836).^8^ Details of the study have been published elsewhere.^5^^,^^8^^,^^26^ In brief, the trial recruited individuals aged 20–65 years who had been newly diagnosed with type 2 diabetes, had a body-mass index of 27–45 kg/m^2^, and were not receiving insulin through GP practices. GP practices were invited to participate in multiple Health Board areas in Scotland and in the Newcastle -upon-Tyne NHS Foundation Trust area in the North East of England by the Primary Care Research Network (PCRN), followed by recruitment by the primary research team (for trail protocol see^26^). Recruitment took place between 25th July 2014 and 5th August 2016. Participants were recruited across 49 primary care practices in Scotland and the Tyneside region of England. The intervention included withdrawal of antidiabetic and antihypertensive drugs. This was followed by a total diet replacement (825–853 kcal/day formula diet (Counterweight-Plus) for 3–5 months), and stepped food reintroduction phase (two to eight weeks), and finally structured support for long-term weight-loss maintenance.^26^ Participants were assigned either to a weight management programme (intervention) or best-practice care by guidelines (control) (see *Randomisation and masking* for more information). Phenotypic data were collected at baseline, 12 months, and 2 years. At 12 months, participants in the intervention arm experienced a mean weight-loss of 10 kg, and 46% of participants were in diabetes remission (defined as glycated haemoglobin of less than 6.5% after at least 2 months off all antidiabetic medications).^8^ Participants in the control arm experienced a mean weight-loss of 1 kg, and 4% of participants were in diabetes remission.^8^ Sex was collected as self-reported by study participants, and the impact of sex on the intervention effects was not investigated due to limited power.

### Randomisation and masking

Practices were randomly assigned (1:1) to provide either a weight management programme (intervention) or best-practice care by guidelines (control), using a minimisation method to maintain balance according to study site (Tyneside or Scotland) and practice list size (>5700 or ≤5700). Cluster randomisation at the GP practice level was performed to avoid contamination between groups and allow consistent advice from practice nurses/dietitians. Randomisation was performed by the Robertson Centre for Bio-Statistics, University of Glasgow, independently of the primary research team. See previous publications for further detail.^5^^,^^8^^,^^26^

### Proteomic data

In total, a subset of 574 available serum samples from 313 participants (260 of whom had paired samples at baseline and 12 months) collected during the trial were analysed using the Olink Proteomics Oncology II assay (Olink Target 96 Oncology II (v.7004); http://www.olink.com). The proteins included in the panel were specifically selected for their relevance to cancer based on public-access bioinformatic databases.^28^ Prior to blood draw, participants were asked to fast overnight. Blood samples were drawn into a serum separator tube and stored at 4 °C before processing. Samples were centrifuged at 2000*g* for 15 min at 4 °C within 5 h of collection. Serum was separated and stored in 0.5 mL aliquots at −80 °C. Olink Target 96 platforms require 40 μL serum and use paired oligonucleotide labelled antibodies to bind and measure the relative abundance of 92 proteins simultaneously using a proximity extension assay.^29^ Samples were sent for analysis by Olink (Uppsala, Sweden). All analysts were blind to intervention/control arm status.

Normalised protein expression (NPX) data were returned by Olink. One NPX unit reflects a doubled protein concentration. Samples deviating >0.3 NPX units from the median were excluded. We applied further quality control (QC) steps using the metaboprep R package: samples and features were excluded based on extreme missingness (80%) before missingness was recalculated and exclusions made using a 20% threshold, additional exclusions were based on sample total sum abundance (>5 SD from the mean) and PCA of independent features (>5 SD from the mean).^30^ Protein measurements below the limit of detection were retained with their recorded value with the caveat that data below the limit of detection have a higher risk to be in the non-linear phase of the S-curve of the NPX unit which may bias estimates. All protein measures were standardised and normalised prior to analyses using rank-based inverse normal transformation (RNT).

### Statistical analysis

#### Evaluation of the effect of the DiRECT intervention on serum protein levels

To evaluate the effect of the intervention on serum protein levels, outcomes (i.e., protein levels at 12 months follow-up) were compared between groups with linear models applied to the RNT data. Where protein measurements at baseline or follow-up were missing (unquantified) for an individual, that individual was excluded from the analysis for that specific protein and therefore, the analysed sample size varied slightly across proteins (N = 258–260). The maximum sample size was 260 paired samples. Models were adjusted for study centre and clinical practice list size along with the baseline measurement of the outcome (protein), age and sex, all fitted as fixed effects. Our primary exposure, analysed for all available data was group (trial arm) allocation; therefore, our estimates reflect the intervention effect in a clinical setting accounting for non-compliance. Effect estimates represent the difference in serum protein level seen in the intervention group relative to the control group while adjusting for protein levels at baseline (on the RNT standard deviation (SD) scale). The Holm method^31^ was used to adjust *P*-values for multiple testing with an adjusted *P*-value of <0.05 used as a heuristic for strong evidence of an effect.

We also examined the observational association between oncology-related proteins and BMI at baseline (N = 260, mean BMI 34.5 kg/m^2^, SD 4.4 kg/m^2^) to interrogate whether intervention effects were likely reflective of changes in body composition or other aspects of the intervention (e.g., dietary exposures). Associations between protein levels and BMI at baseline were estimated using linear regression adjusted for age, sex, study centre and practice list size. Model effect estimates represent the estimated difference in protein level (SD units) per unit change in the respective independent variable.

#### Mendelian randomization and colocalisation analyses of intervention-associated proteins with cancer risk

In addition to the primary trial-based results characterising protein differences associated with randomised intervention, Mendelian randomization and colocalisation analyses were conducted using independent datasets to estimate the causal relationship between intervention-associated (Holm corrected *P*-value <0.05) proteins and colorectal, breast, endometrial, gallbladder, liver, and pancreatic cancer risk.^27^ To obtain genetic instruments for intervention-associated proteins, association estimates for the top *cis* protein quantitative trait loci (pQTL, SNP with the smallest *P* value within 1 Mb from the gene encoding the protein as defined by Sun et al.^32^) passing a multiple-testing corrected threshold of *P* < 3.4 × 10^−11^ were obtained from a genome-wide association study (GWAS) of 1462 circulating proteins detected by the Olink Explore 3072 assay, conducted in up to 54,306 UK Biobank participants. These data are given in NPX values and therefore represent an arbitrary unit on the log2 scale. As such, values are comparable across individuals for the same protein, but not across proteins or across analyses.^32^ Participants who had been diagnosed with blood cancer, or those with leukocyte count >200 × 10^9^/L or >100 × 10^9^/L with 5% immature reticulocytes, haemoglobin concentration >20 g/dL, haematocrit >60%, and platelet count >1000 × 10^9^/L were excluded. Therefore, it is possible that some of the participants included in this GWAS had recently been diagnosed with other cancer types and were undergoing cancer treatment, which could impact their serum protein levels and is therefore a limitation of using this GWAS. However, given the sample size of 54,306 and the exclusion criteria, it is unlikely that these individuals, if present, represent a substantial proportion of the GWAS. pQTLs were functionally evaluated using the Ensembl Variant Effect Predictor (VEP, based on RefSeq and Ensembl).^33^ Suitable genetic instruments were available for eight out of the nine intervention-associated proteins (all except pancreatic prohormone (PPY)).

Summary genetic association data were obtained from six cancer-specific GWAS. Association estimates for colorectal cancer in 52,775 cases and 45,940 controls were obtained from the ColoRectal Transdisciplinary Study (CORECT), the Colon Cancer Family Registry (CCFR), and the Genetics and Epidemiology of Colorectal Cancer (GECCO) consortium. Association estimates provided for the analysis did not include UK Biobank study participants to avoid potential overlap between protein and colorectal cancer summary statistics.^34^ Association estimates for liver cancer were obtained from a GWAS of liver cancer (specifically hepatocyte carcinoma) conducted in up to 648 cases and 259,583 controls in FinnGen.^35^ Summary genetic data for pancreas cancer were obtained from a GWAS of 1249 cases and 259,583 controls in FinnGen.^35^ Summary genetic association data were obtained for breast cancer from a GWAS of 133,384 cases and 113,789 controls of European ancestry.^36^ Summary genetic association data were obtained for endometrial cancer from a GWAS of 12,906 cases and 108,979 controls of European ancestry.^37^ Summary genetic data for gallbladder cancer were obtained from the UK Biobank, which included 195 cases and 456,134 controls of European ancestry.^38^ In the Mendelian randomization analysis of protein levels and gallbladder cancer, both GWAS were obtained using UK Biobank participants. Therefore, there is potential for sample overlap between the studies (i.e., the same individuals being included in both exposure and outcome datasets). Sample overlap in two sample Mendelian randomization analyses can bias estimates towards the confounded observational estimate in the presence of weak instrument bias.^39^ Any analyses with at least weak evidence for an effect of protein levels on gallbladder cancer (*P* < 0.05) were repeated using an alternative GWAS of gallbladder performed in FinnGen participants (84 cases, 259,584 controls), avoiding any sample overlap.^40^

Wald ratio estimates^41^ were calculated to estimate the causal effect of intervention-associated proteins on cancer risk. Summary statistics were harmonised using the harmonise_data function within the TwoSampleMR R package (version 0.5.7).^42^ Three core assumptions must be satisfied for Mendelian randomization analyses to be valid: (i) that the genetic instrument is associated with the exposure under investigation (“relevance”); (ii) that the genetic instrument does not share a common cause with the outcome under investigation (“exchangeability”); (iii) that the genetic instrument has no direct effect on the outcome (“exclusion restriction”).^23^ F-statistics were computed to appraise the “relevance” assumption. We examined F-statistics for evidence of weak instrument bias, defined as an F-statistic less than 10.^43^ We also examined if *cis* variants used to instrument intervention-associated proteins were nominally associated (*P*-value < 0.05) and directionally concordant in a GWAS of proteins detected using a different technique (SomaScan v4 assay).^44^ The online calculator available at https://sb452.shinyapps.io/power/ was used for Mendelian randomization power calculations.^44^

We also performed genetic colocalisation to examine whether proteins and cancer outcomes share the same causal variant at a given locus, which is necessary for (though not alone sufficient for evidence of) a causal relationship between the traits. Colocalisation was performed using the *coloc* R package (version 5.1.0.1).^45^^,^^46^ *coloc* uses approximate Bayes factor computation to estimate posterior probabilities that associations between two traits represent: (i) no genetic association in the region for either trait (H_0_), (ii) a genetic association in the region for the first trait (H_1_), (iii) a genetic association in the region for the second trait (H_2_), (iv) associations for both traits, but different causal variants (H_3_) and (v) associations for both traits with a shared single causal variant (H_4_).^45^ Priors were selected based on a window size of 10 Mb and a p1 of 0.000001, p2 of 0.000001, and p12 of 0.000001 using the online calculator available at: https://chr1swallace.shinyapps.io/coloc-priors/. SomaScan association estimates from Pietzner et al.^44^ were used for colocalisation analyses as full summary statistics were not available from Sun et al.^32^ at the time of publication. Variants within 10 Mb of pQTLs used to instrument serum proteins for Mendelian randomization estimates were included in colocalisation analyses. A posterior probability of >0.80 for H_4_ (i.e., a shared causal variant between the traits) was used to indicate evidence of colocalisation.^47^

### Ethics

DiRECT is registered with the ISRCTN registry, number 03267836. Ethics approval for the DiRECT trial was granted by West 3 Ethics Committee in January 2014 (reference number 13/WS/0314), with approvals by the National Health Service (NHS) health board areas in Scotland and clinical commissioning groups in Tyneside, in accordance with the Declaration of Helsinki. All participants provided informed consent.

### Role of funders

The funders had no role in study design, data collection, data analyses, interpretation, or writing of the manuscript.

## Results

### Effect of the weight-loss intervention on serum proteins

**Fig. 1:**
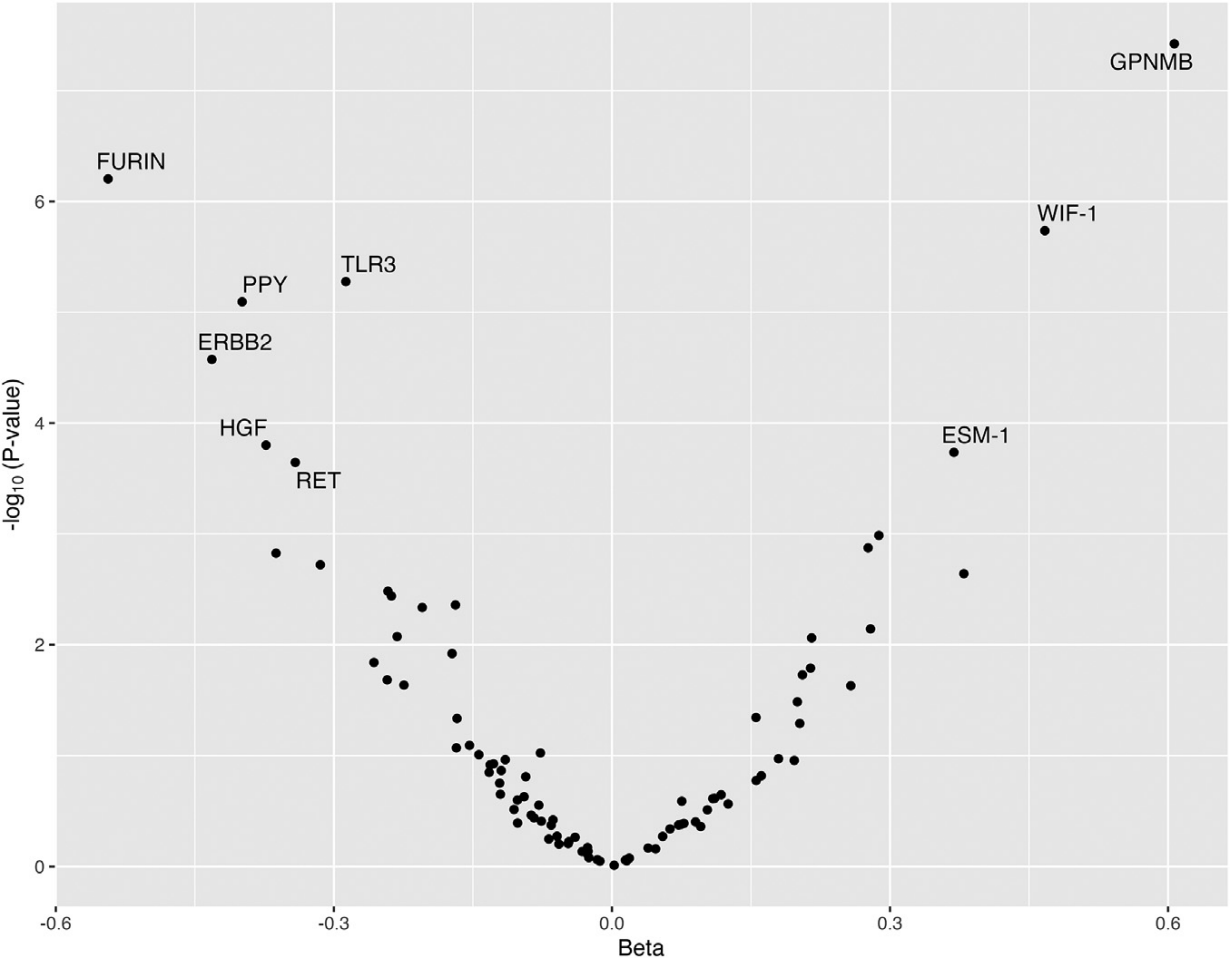
Comparison of serum protein levels at 12 months follow-up between intervention groups (n = 260). Betas reflect the mean difference between allocation groups (protein measures were standardised and normalised prior to analyses using rank-based inverse normal transformation) while adjusting for protein levels at baseline. Labelled proteins are those that pass a Holm corrected *P* = 0.05. Values greater than 0 indicate an increase in serum protein seen in the intervention arm and values less than 0 reflect a decrease in serum protein levels. GPNMB: Glycoprotein Nmb; FURIN: Furin; WIF1: WNT Inhibitory Factor 1; TLR3: Toll-like receptor 3; PPY: Pancreatic prohormone; ERBB2: Receptor tyrosine-protein kinase erbB-2; HGF: Hepatocyte growth factor; ESM-1: Endothelial cell-specific molecule 1; RET: Proto-oncogene tyrosine-protein kinase receptor Ret.

Following data QC, 260 participants (117 intervention, 143 control, Supplementary Table S1) had trial and proteomic data available measured in samples at baseline and 12 months. The minimum sample size for the analysis of an individual protein was 258. A large proportion of samples (57%) were below the level of detection for one protein (FAS-associated death domain protein, FADD). Baseline characteristics were similar between the control and intervention groups (Table 1). Overall, 9 proteins were modified by the intervention in the linear model analysis (Holm corrected *P*-value <0.05)—3 of which were increased, and 6 reduced by the intervention (Fig. 1 and Table 2, full results Supplementary Table S2). The strongest evidence for a positive association was seen for glycoprotein Nmb (GPNMB, Beta: 0.61, SE: 0.11, Holm corrected *P*-value = 3.78e-08) with 8% of variance in glycoprotein Nmb at 12 months explained by group allocation. The strongest evidence for a negative association was seen for furin (FURIN, Beta: −0.54, SE: 0.11, Holm corrected *P*-value = 6.28e-07), with 7% of the variance in furin at 12 months explained by group allocation. Of the 9 intervention-associated proteins, 5 were associated with BMI at baseline in a direction consistent with the effect of weight-loss on the proteins (*P* < 0.05; furin, hepatocyte growth factor, toll-like receptor 3, receptor tyrosine-protein kinase erbB-2 and proto-oncogene tyrosine-protein kinase receptor Ret, Supplementary Table S3).

**Table 1:** Baseline characteristics of participants with type 2 diabetes included in the study (N = 260).

	Intervention group (n = 117)		Control group (n = 143)	
	Mean/N	SD/%	Mean/N	SD/%
Sex				
Female	49	42%	55	38%
Male	68	58%	88	62%
Age (years)	53.7	7.1	56.3	6.8
Female	54.0	7.0	56.5	7.0
Male	53.5	7.0	56.3	7.0
Body mass index (kg/m^2^)	34.8	4.5	34.3	4.3
Female	35.7	4.4	34.8	4.4
Male	34.2	4.4	34.0	4.4
Weight (kg)	100.3	16.9	98.9	16.3
Female	91.4	16.3	90.4	16.3
Male	107.0	16.3	104.0	16.3
Fasting glucose (mmol/L)	9.3	3.2	8.8	2.6
Female	9.6	2.9	8.7	2.9
Male	9.1	2.9	8.9	2.9
Total cholesterol (mmol/L)	4.3	1.1	4.3	1.1
Female	4.7	1.1	4.4	1.1
Male	4.0	1.1	4.2	1.1
HDL cholesterol (mmol/L)	1.1	0.3	1.2	0.3
Female	1.2	0.3	1.3	0.3
Male	1.1	0.3	1.1	0.3
Triglycerides (mmol/L)	2.0	1.5	1.9	0.9
Female	1.8	1.2	1.8	1.2
Male	2.2	1.2	2.1	1.2

Summary statistics based on the analysis sample (N = 260). SD = standard deviation.

**Table 2:** Association of serum protein levels and intervention status at 12 months follow-up.

Protein ID	Protein name	Beta	SE	*P*-value	Adjusted *P*-value
GPNMB	Glycoprotein Nmb	0.61	0.11	3.78e-08	3.48e-06
FURIN	Furin	−0.54	0.11	6.28e-07	5.71e-05
WIF1	WNT Inhibitory Factor 1	0.47	0.10	1.84e-06	1.66e-04
TLR3	Toll-like receptor 3	−0.29	0.06	5.30e-06	4.72e-04
PPY	Pancreatic prohormone	−0.40	0.09	8.06e-06	7.10e-04
ERBB2	Receptor tyrosine-protein kinase erbB-2	−0.43	0.10	2.66e-05	2.32e-03
HGF	Hepatocyte growth factor	−0.37	0.10	1.58e-04	0.01
ESM-1	Endothelial cell-specific molecule 1	0.36	0.10	1.84e-04	0.02
RET	Proto-oncogene tyrosine-protein kinase receptor Ret	−0.34	0.09	2.26e-04	0.02

Betas reflect the association of intervention group with in serum protein levels at 12 months follow-up while adjusting for protein levels at baseline (protein measures were standardised and normalised prior to analyses using rank-based inverse normal transformation).

Adjusted *P*-value refers to a Holm corrected *P*-value. Listed proteins are those that pass a Holm corrected *P* = 0.05.

### Effect of modified proteins on cancer risk

Details of genetic variants used to instrument oncology-related proteins are presented in Supplementary Table S4. A single genome-wide significant *cis* pQTL was available for eight of the nine intervention-associated proteins (no genome-wide significant associations were detected for Receptor tyrosine-protein kinase erbB-2 (ERBB2) by Sun et al.^32^). For one of these proteins (furin), the *cis* pQTL was borderline nominally associated (*P*-value = 0.05) in a GWAS of proteins detected using a different technique (SomaScan v4 assay), suggesting possible proxying of binding effects.^44^ Using the Ensembl Variant Effect Predictor it was estimated that the selected pQTLs for *GPNMB, FURIN, TLR3* and *HGF* had consequential effects on gene transcripts (Supplementary Table S4). The minimum F-statistic for protein genetic instruments was 166, suggesting analyses should avoid weak instrument bias (Supplementary Table S4). Power to detect an odds ratio (OR) of 1.2 was >80% for five out of six cancers for at least one protein (all except gallbladder cancer; mean power = 65%; median power = 95%, per SD change oncology-related protein (α = 0.05)) (Supplementary Table S5).

A Bonferroni correction was applied to correct for multiple testing across genetic analyses, resulting in a *P*-value threshold of < 0.001 (0.05/48 statistical tests; eight proteins against six cancer end points) with this threshold being used to define “strong evidence”. Findings where *P* ≥ 0.001 and *P* < 0.05 were interpreted as “weak evidence”.

**Fig. 2:**
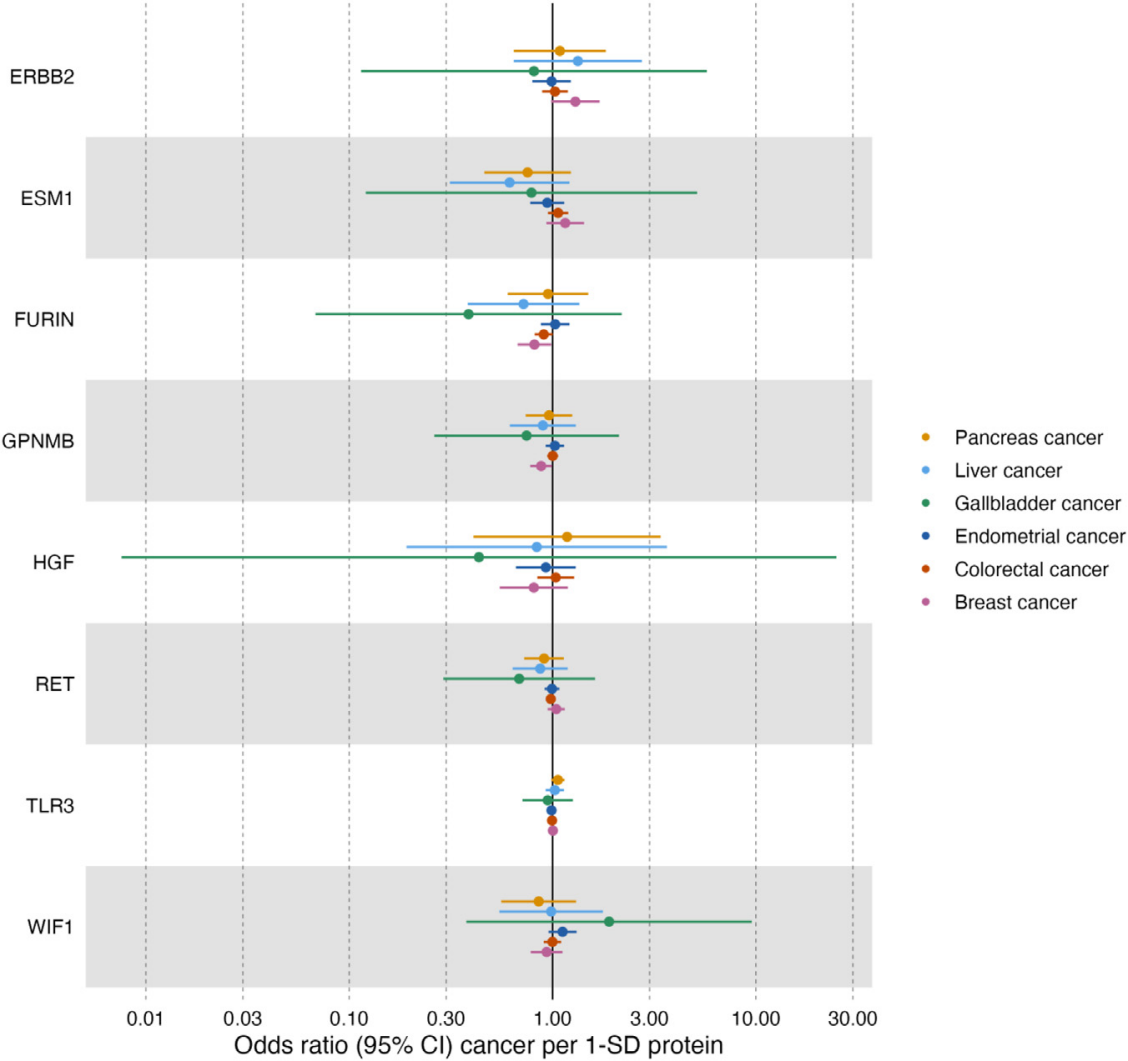
Estimated effect of circulating proteins on cancer risk in Mendelian randomization analyses (odds ratio (OR) and 95% confidence interval; n = 98,715–260,832 depending on cancer). All analyses used the Wald ratio model. Where estimates are missing the genetic instruments for the exposure were not present in the outcome dataset and thus MR could not be performed. GPNMB: Glycoprotein Nmb; FURIN: Furin; WIF1: WNT Inhibitory Factor 1; TLR3: Toll-like receptor 3; PPY: Pancreatic prohormone; ERBB2: Receptor tyrosine-protein kinase erbB-2; HGF: Hepatocyte growth factor; ESM-1: Endothelial cell-specific molecule 1; RET: Proto-oncogene tyrosine-protein kinase receptor Ret; CI = confidence interval.

Amongst all protein to cancer associations investigated, there was weak evidence to support an effect of furin and glycoprotein Nmb on cancer risk (Fig. 2 and Supplemental Table S6). We found weak evidence that increased serum furin reduces breast cancer risk (OR 0.81, 95% CI = 0.67–0.99, *P* = 0.03 per SD increase in serum furin), and that increased glycoprotein Nmb reduces breast cancer risk (OR 0.88, 95% CI = 0.78–0.99, *P* = 0.04 per SD increase in serum glycoprotein Nmb). Genetic colocalisation analysis suggested that serum furin and glycoprotein Nmb were unlikely to share a causal variant with risk of breast cancer (posterior probability of a shared causal variant (H_4_): ≤80%, Supplemental Table S7).

## Discussion

There is strong evidence that individuals with type 2 diabetes are at increased risk of at least six cancer types.^1^ Multiple studies have investigated the influence of obesity on circulating intermediate traits, but few studies have examined the impact of weight-loss on circulating proteins, particularly in individuals diagnosed with type 2 diabetes, or how circulating proteins relate to type 2 diabetes-related cancer risk. In this study, we investigated the impact of a type 2 diabetes weight-loss intervention (Counterweight-Plus) on oncology-related proteins in serum. Under a conservative correction for multiple testing, nine proteins were associated with intervention status (weight-loss): glycoprotein Nmb, furin, Wnt inhibitory factor 1, toll-like receptor 3, pancreatic prohormone, erb-b2 receptor tyrosine kinase 2, hepatocyte growth factor, endothelial cell specific molecule 1, and Ret proto-oncogene. We then evaluated these proteins for an observational association with BMI at baseline, and for evidence of a causal effect on risk of type 2 diabetes-related cancers in Mendelian randomization analyses.

Although the proteins investigated were specifically selected for their biological relevance to cancer,^28^ the majority of proteins associated with the weight-loss intervention in this study are typically membrane bound or intracellular and therefore the relevance of serum levels of these proteins to cancer risk is less well known. Of the 92 proteins investigated, glycoprotein Nmb, a transmembrane glycoprotein, showed the largest increase in response to weight-loss, but was not associated with BMI at baseline suggesting the observed association may not be driven by BMI specifically and may reflect another aspect of the intervention e.g., dietary exposures. Our Mendelian randomization and colocalisation analyses found little evidence for a causal role for serum glycoprotein Nmb in development of six type 2 diabetes-related cancers (colorectal, breast, endometrial, gallbladder, liver, and pancreatic cancers), though the tissue-specific relevance or predictive utility of serum glycoprotein Nmb in type 2 diabetes-related cancer risk should be further investigated.

A recent pan-cancer analysis reported that increased expression of furin in cancer tissue was associated with poor prognosis.^48^ Consistent with a protective effect of weight-loss on cancer risk, we found that serum furin was reduced following the Counterweight Plus intervention and that furin was positively associated with BMI at baseline. However, our Mendelian randomization analyses suggested there might be a protective effect of higher serum furin on breast cancer risk, which would suggest a possible detrimental effect of this reduction in the intervention group. Colocalisation analyses, however, suggested serum furin was unlikely to share a causal variant with breast cancer in this region given low posterior probabilities to support H_4_ (i.e., shared causal variants). The lack of colocalising signal and estimated protective effect of serum furin on cancer risk in genetic analyses may be attributable to our ability to reliably instrument (proxy) serum furin with a single *cis* SNP (rs6227), a reduced signal in the furin locus in the GWAS of proteins measured using the SomaScan platform,^44^ or potential genetic pleiotropy of the variant (this SNP has also been found to influence expression of FES proto-oncogene, tyrosine kinase.)^49^

Circulating levels of toll-like receptor 3, another transmembrane protein, were also found to be reduced with weight-loss. Consistent with the effect of the intervention, we found that toll-like receptor 3 was positively associated with BMI at baseline. Across solid tumours from differing tissues, toll-like receptor 3 expression has been found to be associated with both good and poor prognosis in cancer, possibly via pro-apoptotic pathways and resistance to anti-tumour drugs, respectively.^50^ In Mendelian randomization analyses, we observed little evidence for a causal effect of increased serum toll-like receptor 3 on any cancer risk.

In the trial analysis, we found that pancreatic prohormone, receptor tyrosine-protein kinase erbB-2, hepatocyte growth factor and proto-oncogene tyrosine-protein kinase receptor Ret were reduced with weight-loss. Of these proteins, hepatocyte growth factor and tyrosine-protein kinase receptor Ret were positively associated with BMI at baseline. Our results for hepatocyte growth factor are consistent with previous cross-sectional studies which have reported a positive correlation between obesity and hepatocyte growth factor in serum.^51^^,^^52^ Hepatocyte growth factor is known to promote proliferation, migration, invasion and survival of cancer cells.^53^ However, the origin of hepatocyte growth factor present in the tumour microenvironment is unknown as it may be synthesised by a variety of tissues throughout the body, including liver or adipose tissue. Mendelian randomization and colocalisation estimates did not support evidence of causality between hepatocyte growth factor and type 2 diabetes-related cancers.

In the trial analysis we found that serum Wnt Inhibitory Factor 1 and endothelial cell-specific molecule 1 (endocan) were increased with weight-loss, though these proteins were not consistently associated with BMI at baseline and follow-up genetic analyses did not identify any effects of serum Wnt Inhibitory Factor 1 or endothelial cell-specific molecule 1 on type 2 diabetes-related cancer risk. Our findings are inconsistent with previous cross-sectional studies that have reported a positive correlation between obesity and serum levels of endothelial cell-specific molecule 1.^54^^,^^55^ It is possible these earlier estimates may be influenced by residual confounding which should be minimised in our intervention-based approach.

One limitation of our analysis is that we were unable to ascertain whether the intervention-related changes identified were due directly to weight-loss, to the rapid dietary change, to the withdrawal of antidiabetic and antihypertensive medication, or as a result of the food reintroduction stage. However, an association between protein levels and BMI at baseline may be indicative of a driving effect of an association between protein levels and adiposity. Of the nine intervention-associated proteins, there was strong evidence (*P* < 0.05) for five of the proteins for an association with BMI at baseline (furin, hepatocyte growth factor, toll-like receptor 3, receptor tyrosine-protein kinase erbB-2 and proto-oncogene tyrosine-protein kinase receptor RET). This replicates results from a previous analysis using SomaLogic proteomic data from up to 2737 healthy participants other than for toll-like receptor 3, which was not included in the previous analysis, and receptor tyrosine-protein kinase erbB-2, for which they found little evidence of an association (*P* > 0.05).^17^ In our analysis, the direction of the association was consistent with the direction of effect of the intervention (i.e., if the intervention decreased protein levels, the association with BMI at baseline was positive and vice versa) for a further protein (Glycoprotein Nmb), despite the *P*-value not reaching the predefined cut-off of 0.05. In the previous analysis, Glycoprotein Nmb showed weak evidence for an association with BMI (*P* < 0.05) in a direction consistent with our analyses, suggesting that we may not have had sufficient sample size to detect this association. Three proteins showed an inconsistent direction of effect between the weight-loss intervention and BMI at baseline (WNT inhibitory factor 1, pancreatic prohormone, and endothelial cell-specific molecule 1). For WNT inhibitory factor 1 and endothelial cell-specific molecule 1, the previous analysis found strong evidence (*P* < 1.4 × 10^−5^) for an association with BMI in a direction consistent with the effect of the weight-loss intervention in our analysis.

One possible explanation for the lack of evidence for an association with BMI at baseline in our analysis is that the relationship between BMI and protein levels may be non-linear, so the association may not be observable in a population where BMI is ubiquitously high. For the final intervention-associated protein (pancreatic prohormone), the previous analysis found strong evidence (*P* < 1.4 × 10^−5^) for an association with BMI in the same direction to that in our analysis, i.e., in a direction inconsistent with the direction of association with the intervention. This suggests that the effect of the intervention may not be driven by change in BMI itself but another aspect (e.g., a non-weight-related response to dietary change). Recent work has highlighted that some aspects of adiposity which are relevant to health outcomes may not be captured by BMI. For instance, body fat distribution has been shown to have differential effects on cardiovascular outcomes depending on anatomical location of adipose tissue.^56^^,^^57^ Additionally, some differential effects have been seen in the relationship between BMI and cancer risk according to sex.^19^^,^^58^ Although such measurements were not available in this analysis, future work should evaluate the role of body fat distribution in altering oncology-related protein levels in serum, and whether these effects are likely to differ by sex.

We found little evidence for a shared causal variant between expression of any of the proteins and risk of cancers investigated in colocalisation analyses, including for those proteins which had evidence for an effect in Mendelian randomization analyses. One possible explanation for this is a lack of true causal relationships across expression of the nine proteins and six cancer sites investigated. For instance, in the case of furin, the pQTL used to proxy expression in the Mendelian randomization analyses, rs6227, is more strongly associated with expression of neighbouring protein FES proto-oncogene, tyrosine kinase than furin.^49^ Thus, it is possible that the effect seen in this and other Mendelian randomization analyses could in fact reflect a causal association of neighbouring genes on cancer risk rather than the protein being instrumented. However, there are several other explanations for a lack of strong evidence of colocalisation between expression of the proteins investigated here and cancer risk. Firstly, due to a lack of available full summary statistics in the Sun et al. GWAS,^32^ we used an alternative GWAS for the colocalisation analyses which used SomaScan technology to quantify proteins. One limitation of this approach is that several cis pQTLs used to proxy protein levels in the Mendelian randomization analyses are missense variants, which have been well-documented to increase the risk of aptamer binding effects in the SomaScan technology. Secondly, it is likely that the reason for a lack of shared causal variant between exposure and outcome is due to lack of power from sample size in the outcome (i.e., cancer) datasets, which may be limiting the reliability of the colocalisation analyses described here.

A major strength of this study is the use of sequential samples and clinical data from a large and well characterized clinical trial (DiRECT).^5^^,^^8^^,^^26^ We measured 92 oncology-related proteins in paired samples from the same participants at baseline and 12 months. Though it was not possible to measure proteins in all participants, this subset with samples reflects the original trial population, which has been shown to be representative of the general population of people with short duration type 2 diabetes.^59^ Due to the importance of potential pathways linking type 2 diabetes and adiposity with cancer risk, we chose to measure proteins with a known link with cancer determined through bioinformatic databases (i.e., those included in Olink's oncology II panel) which limits our ability to identify novel cancer-relevant proteins. Further, as proteins are measured on an arbitrary scale, this limits our ability to assess the impact of the DiRECT intervention on protein concentrations in absolute units. Future studies should explore the use of novel technologies such as Olink Flex^60^ which can be used to translate NPX values of certain proteins to concentrations in pg/mL units which are more meaningful. Whilst our study design enabled us to confirm that the Counterweight-Plus intervention influences the levels of oncology-related proteins in serum, it is challenging to attribute changes to specific elements of the intervention (e.g., weight-loss or dietary exposures). We attempted to appraise weight-loss specific effects by performing a cross-sectional analysis of protein levels and BMI at baseline, however, this analysis may be underpowered to detect weak effects given the narrow BMI range of participants recruited by DiRECT, which may also make detection of non-linear associations challenging. Another limitation of our analysis is that in limiting the analysis to one cohort, the transferability of our findings to other groups is as of yet unknown. To attempt to extend these results beyond the trial population, we conducted Mendelian randomization and colocalisation analyses using independent datasets in order to estimate the causal relationship between intervention-associated proteins and the risk of six cancers where type 2 diabetes is an important cause (pancreas, colorectal, breast, gallbladder, endometrial and liver cancer).^27^ As we selected only the top *cis* variants to instrument proteins, given that *trans*-acting variants are typically considered to be more prone to introducing horizontal pleiotropy into analyses, it was not possible to conduct sensitivity analyses which require a greater number of genetic variants such as MR-Egger or weighted median methods.^61^^,^^62^ Finally, for one of the nine intervention-associated proteins, pancreatic prohormone (PPY), no suitable genetic instruments were available, meaning we could not include this protein in our Mendelian randomization analyses.

In summary, we performed an analysis of samples from a weight-loss intervention trial and found that nine cancer-related proteins were associated with intervention status (glycoprotein Nmb; furin; Wnt inhibitory factor 1; toll-like receptor 3; pancreatic prohormone; erb-b2 receptor tyrosine kinase 2; hepatocyte growth factor; endothelial cell specific molecule 1 and Ret proto-oncogene). These changes provide evidence that weight management in individuals recently diagnosed with type 2 diabetes delivered in a primary care setting may influence biological pathways relevant to cancer. Follow-up Mendelian randomization analyses provided weak evidence for a causal role of 2 of these proteins in type 2 diabetes-related cancer, specifically furin and glycoprotein Nmb in breast cancer risk. Further evaluation of proteins associated with weight-loss could help to facilitate the development of cancer prevention strategies.

## Contributors

Conceptualisation: R.T., N.S., N.J.T., E.E.V Methodology: C.J.B, L.J.C., D.A.H., J.Y., T.G.R, E.H. Sample preparation: D.N.L., E.E.V. Formal Analysis, C.J.B., E.H. Resources, E.E.V., N.S., R.T., M.E.J.L. Writing–Original Draft: C.J.B., E.H.; All authors reviewed, contributed to, and approved the final version of the manuscript; Supervision: N.J.T. and E.E.V Funding Acquisition, R.T., N.S., M.E.J.L., N.J.T and E.E.V.; Data collection: A.B.H. E.H. and C.J.B. have verified the underlying data.

## Data sharing statement

Due to the sensitive nature of the data used in this study, requests to access the data should be made to the Principal Investigators of the DiRECT trial via https://www.directclinicaltrial.org.uk/DiRectStudyTeam.html. Summary statistics used for MR analyses were obtained from the reference GWAS. R scripts used in this study have been made publicly available on GitHub at: https://github.com/cb12104/direct_olink_oncology. All analyses were performed in R version 4.0.3.^63^

## Declaration of interests

Tom G Richardson is an employee of GlaxoSmithKline outside of the research presented in this manuscript. Mike Lean has received lecturing fees from Novo Nordisk, Roche, Merck, Sanofi Nestle and Oviva, recognises grants from Diabetes UK, NIHR, and All Sants Educational Trust, and consulting fees from Counterweight. Roy Taylor has received lecture honoraria from Eli Lilly, Nestle Health and Janssen and payment or honoraria from educational videos for European Association for the Study of Diabetes, and recognises grant support from Diabetes UK. Alex McConnachie recognises grant support from Diabetes UK. Emma Hazelwood recognises support for travel from the Harold Hyam Wingate Foundation, the European Cancer Prevention organization, and the European Association for Cancer Research, and sits on the IGES Ethical, Legal and Societal Issues committee. Naveed Sattar recognizes grant support from AstaZeneca, Boehringer Ingelheim, Novartis, and Roche Diagnostics, has received consulting fees from Abbott Laboratories, Amgen, AstraZeneca, Boehringer Ingelheim, Eli Lilly, Hanmi Pharmaceuticals, Janssen, Marck Sharp & Dohme, Novartis, Novo Nordisk, Pfizer, Roche Diagnostics, and Sanofi, and has received payment for lectures or manuscript writing from Abbott Laboratories, AstraZeneca, Boehringer Ingelheim, Eli Lilly, Janssen, and Novo Nordisk. Paul Welsh recognizes grant support from AstraZeneca, Roche Diagnostics, Boehringer Ingelheim, and Novartis, and payment for lectures or manuscript writing from Novo Nordisk and Raisio Nutrition. Rachel Pearlman is an executive council member of CGA-IGC.

The remaining authors declare no competing interests.

Where authors are identified as personnel of the International Agency for Research on Cancer/World Health Organization, the authors alone are responsible for the views expressed in this article and they do not necessarily represent the decisions, policy or views of the International Agency for Research on Cancer/World Health Organization.
